# Myoepithelial carcinoma with RB1 mutation: remarkable chemosensitivity to carcinoma of unknown origin therapy

**DOI:** 10.1186/s12885-017-3249-x

**Published:** 2017-04-08

**Authors:** Timothy M. Hoggard, Evita Henderson-Jackson, Marilyn M. Bui, Jamie Caracciolo, Jamie K. Teer, Sean Yoder, Odion Binitie, Ricardo J. Gonzalez, Andrew S. Brohl, Damon R. Reed

**Affiliations:** 1 University of South Florida Morsani College of Medicine, 12901 Bruce B Downs Blvd., Tampa, FL 33612 USA; 2 Department of Anatomic Pathology, 12901 Bruce B Downs Blvd., Tampa, FL 33612 USA; 3 Department of Diagnostic Imaging, 12901 Bruce B Downs Blvd., Tampa, FL 33612 USA; 4 Department of Biostatistics and Bioinformatics, 12901 Bruce B Downs Blvd., Tampa, FL 33612 USA; 5 Molecular Genomics Core Facility, 12901 Bruce B Downs Blvd., Tampa, FL 33612 USA; 6 Sarcoma Department, 12901 Bruce B Downs Blvd., Tampa, FL 33612 USA; 7 Chemical Biology and Molecular Medicine Program, 12901 Bruce B Downs Blvd., Tampa, FL 33612 USA; 8 Adolescent and Young Adult Program; H. Lee Moffitt Cancer Center and Research Institute, 12901 Bruce B Downs Blvd., Tampa, FL 33612 USA

**Keywords:** Myoepthelioid carcinoma, RB1, Chemotherapy, Paclitaxel, Carboplatin

## Abstract

**Background:**

Myoepithelial carcinoma of soft tissue is a rare, malignant neoplasm that is morphologically and immunophenotypically similar to its counterpart in salivary gland. It demonstrates myoepithelial differentiation, possessing both epithelial and myogenic characteristics. Thought to be chemotherapy insensitive, the optimal treatment regimen of this tumor has yet to be established and only a select few cases in the literature discuss treatment efficacy in detail.

**Case presentation:**

Here we present a case of a young adult with metastatic myoepithelial carcinoma with an initial excellent response to systemic therapy utilizing carboplatin and paclitaxel with continued complete response after 3 years. The patient also underwent complete surgical excision and received adjuvant radiation to the primary site of disease. Exome sequencing revealed an inactivating mutation in *RB1* which we believe to be the first such mutation to be reported in this cancer type.

**Conclusions:**

Given increasing evidence suggesting RB1 loss is associated with responsiveness to conventional chemotherapies, particularly platinum-based regimens, we hypothesize that this genetic feature predisposed chemosensitivity in our patient’s tumor.

## Background

Myoepithelial tumors are rare salivary gland tumors classically found in the parotid gland. Most are benign myoepitheliomas. The malignant counterpart, myoepithelial carcinoma, is even more rare and represents less than 2% of salivary gland carcinomas [1]. Most cases of myoepithelial carcinoma are de novo in origin but may occasionally arise in association with a preexisting myoepithelioma or benign mixed tumor (pleomorphic adenoma) [2]. These malignant tumors also occur in non-salivary sites, such the nasopharynx, lung, breast, and skin [3–6]. About 50 cases of soft tissue locations of this tumor, both benign and malignant, have been described most often located in deep subcutaneous, intramuscular, or subfascial tissue of the limbs and limb girdles [1, 6–10]. Compared to their salivary equivalent these tumors demonstrate increasing tendency for metastasis as well as aggressive histologic features, particularly within the pediatric population [1, 11]. These tumors exhibit a heterogenous histomorphology and variable immunophenotypic findings, in turn, proving difficult to diagnosis [1]. Several recurrent molecular underpinnings unique to soft tissue myoepithelial carcinoma have been described, including *EWSR1* gene rearrangements in up to 45% of cases [12]. Additionally, homozygous deletion of *SMARCB1* has been reported in 3/5 cases that lack the *EWSR1* gene rearrangement [13]. Comprehensive molecular analysis of this rare tumor type, however, has not been performed.

## Materials and methods

A chart review was conducted under IRB approval (MCC15003, University of South Florida IRB). To further evaluate our patient for a potential molecular explanation for dramatic chemotherapy response, we performed whole exome sequencing on the initial left popliteal mass resection, prior to any radiation or chemotherapy. Paired-end sequencing was performed on Illumina NextSeq 500 (76 × 2) instrument, generating 214,044,758 total read pairs, resulting in 107× mean coverage across the capture region after duplication removal and mapping. 99.6% of targeted bases achieved at least 10× depth of coverage. Burrows-Wheeler Aligner was used to align sequence reads to the human reference [14]. The Genome Analysis Toolkit was used for insertion/deletion realignment, quality score recalibration, and identification of single nucleotide and insertion/deletion variants [15]. To enrich for somatic mutations, we restricted our analysis to variants that are rare or absent in population databases (MAF <0.01 in 1000 Genomes Project, the NHLBI Exome Sequencing Project, and ExAC database). To further limit our findings to those most likely to be oncogenic, we utilized curated databases including COSMIC and the Cancer Gene Census to manually review variants for functional consequence and known status as an oncogene/tumor suppressor gene.

## Case presentation

A 34-year-old male presented to our institution for evaluation of a left popliteal mass that was present and growing over 1 year with increasing pain. There was no neurologic or vascular compromise distal to the lesion. The patient developed inguinal pain 1 month prior to presentation. Otherwise the review of systems was negative.

**Fig. 1:**
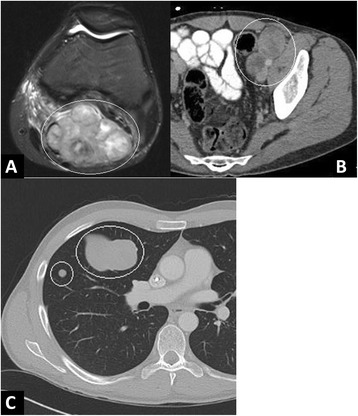
Radiologic presentation. Upon initial presentation, (**a**) axial MRI (short tau inversion recovery/STIR) demonstrate a large lobulated soft tissue mass within the popliteal fossa, (**b**) axial contrast-enhanced CT images demonstrate bulky, necrotic left external iliac lymph nodes, and (**c**) axial CT images demonstrate a dominant *right* lung mass and small nodule consistent with pulmonary metastases

Left knee MRI demonstrated a large, lobulated nonspecific T2-weighted hyperintense soft tissue mass in the popliteal fossa with local mass effect and surrounding soft tissue edema suspicious for soft tissue sarcoma (Fig. 1a). Contrast-enhanced computed tomography of the chest, abdomen, and pelvis performed for tumor staging demonstrated evidence of necrotic left external iliac lymphadenopathy (Fig. 1b), along with a right lung mass and a pulmonary nodule (Fig. 1c) most consistent with distant metastatic disease.

**Fig. 2:**
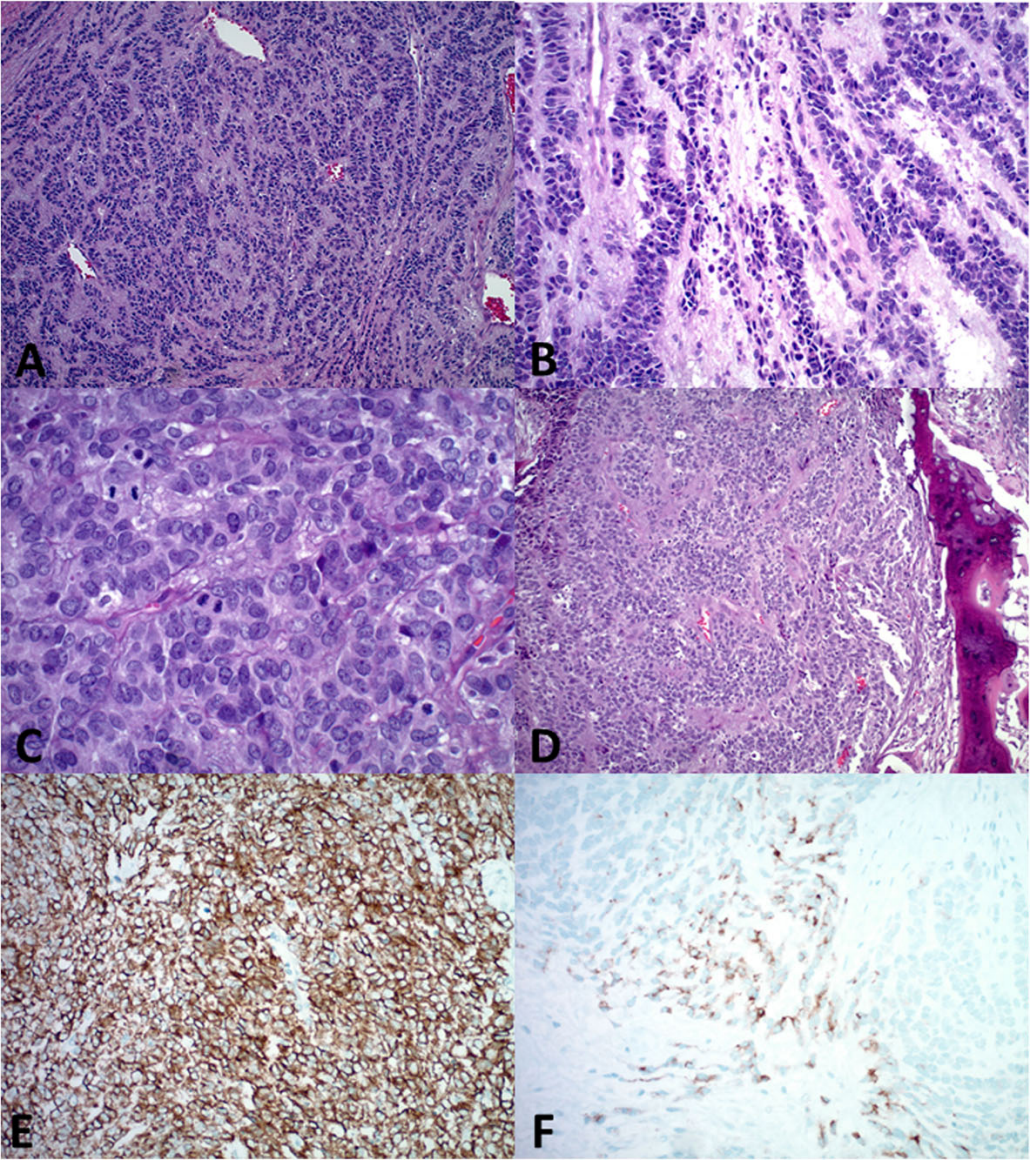
Histologic analysis of tumor specimen at (**a**) 10× and (**b**) 20× magnification demonstrates rounded epithelioid to spindle shaped cells arranged in a trabecular-like fashion. (**c**) High power field demonstrates mitotic activity. (**d**) Bone formation is also noted. (**e**) Immunohistochemical analysis at 20× magnification reveals CKAE1/3 and CAM5.2 reactivity in addition to (**f**) focal EMA reactivity

Tumor cells obtained from CT-guided core biopsy of the popliteal mass and then subsequently of the inguinal lymph nodes showed a proliferation of rounded epithelioid to spindle shaped cells with hyperchromatic nuclei arranged in trabecular-like architecture within hyalinized stroma. Ultimately, complete surgical resection of the primary, popliteal site was performed. Immunohistochemical evaluation revealed reactivity for vimentin, CAM5.2 as well as focal reactivity for CKAE1/3, EMA and synaptophysin. The tumor was negative for S-100, desmin, chromogranin and CD45. Further immunohistochemical analysis following external consultation revealed pankeratin and focal EMA positivity while staining for GFAP, calponin, p63, CD99, FLI-1, CD34, MUC-4, ERG and TLE-1 was negative. Given the limited sample, the lesion was tentatively termed “atypical spindle and round cell neoplasm, possibly myoepithelial in type” (Fig. 2a-f). The patient underwent radical resection of the popliteal mass with a positive margin allowing sufficient tissue to confirm the diagnosis. Grossly, the tumor measured 9.0 × 7.8 × 5.0 cm and cut sections showed an encapsulated, pale white, rubbery, lobulated mass. Histopathologic examination revealed a lobulated, multinodular, infiltrative malignant neoplasm composed of cellular nodules of epithelioid tumor cells with hyperchromatic nuclei showing frequent mitoses arranged in a trabecular fashion. Small proportions of the nodules were hypocellular with tumor cells exhibiting less nuclear atypia and more prominent myxoid stroma. Tumor necrosis was present. The specimen was again sent for consultation and the staining profile mirrored that of the biopsy specimens, aside from focal desmin positivity. Molecular analysis was notably negative for rearrangement of *EWSR1* (22q12) locus and rearrangement of SS18 (SYT; 18q11.2) locus. Additional molecular testing (FISH analysis) performed revealed no rearrangement of NR4A3. In view of the histomorphologic features and reactivity for epithelial markers, a final diagnosis of high-grade myoepithelial carcinoma was rendered both locally and by outside consultation, although the immunophenotype was not definitive in that regard.

**Fig. 3:**
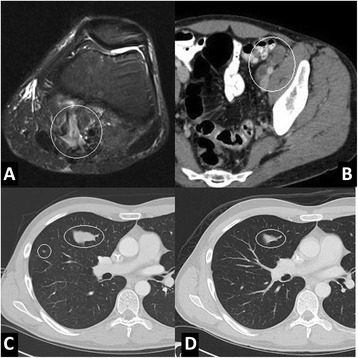
Radiologic response to cisplatin and paclitaxel. **a** Axial MRI demonstrates postoperative changes following surgical resection of popliteal mass without evidence of residual disease. **b** Axial contrast-enhanced CT images following neoadjuvant chemotherapy demonstrate decreased size of left external iliac nodes, consistent with response to therapy. On chemotherapy at (**c**) 1 month and (**d**) 4 months after presentation, both the right lung mass and nodule have markedly improved consistent with response to therapy

Because of the systemic disease burden and limited reported activity of traditional sarcoma chemotherapeutic regimens, the case was discussed amongst medical and pediatric oncologists within and outside our institution without a clear consensus. We elected to treat with 3 cycles of carboplatin and paclitaxel initially with an almost immediate clinical response. Surveillance CT imaging of the chest, abdomen and pelvis demonstrated decreased size of iliac lymph nodes and pulmonary metastases consistent with tumor response to neoadjuvant therapy while MRI demonstrated surgical changes without clear, active disease (Fig. 3a-d).

The patient underwent a completion lymphadenectomy of the left superficial femoral and deep pelvic nodes without evidence of residual tumor in 25 examined lymph nodes. The patient received an additional 2 cycles of carboplatin and paclitaxel. Due to incomplete radiographic response the patient underwent a wedge resection, which also confirmed pathologic complete remission without malignancy identified. Hemorrhage and areas containing epithelioid macrophages with foamy and/or hemosiderin laden cytoplasm along focal adjacent hyaline fibrosis were seen. This was interpreted to be compatible with chemotherapy effect. The patient received adjuvant radiation therapy with 2 Gy fractions ×33 doses to the popliteal fossa. The patient remains in radiographic remission 36 months from completion of chemotherapy. Molecular studies were undertaken to elucidate the mechanism responsible for the durable response to systemic therapy.

## Results

From whole exome sequencing, we identified 509 high-confidence coding variants in our tumor specimen, including 45 truncating (missense, frameshift, or splice site) and 464 nonsynonymous. Of these, we identified 2 truncating mutations in well-described tumor suppressor genes, *RB1* and *MED12*. We additionally note several mutations of less clear oncogenic consequence, including a truncating mutation in *ITGA2*, a possible tumor suppressor, and a nonsynonymous mutation in *PDGFRA* that has been reported in several cancer cases, but is also an uncommon population polymorphism [16–18]. Further description of these notable mutations is provided in Table 1.

**Table 1:** Notable mutations in Myoepithelial Carcinoma tumor sample

Gene	Mutation	dbSNP
*RB1*	NM_000321:exon1:c.38_66del:p.A13fs	-
*MED12*	NM_005120:exon13:c.C1861T:p.R621X	-
*ITGA2*	NM_002203:exon17:c.G2155 T:p.E719X	-
*PDGFRA*	NM_006206:exon5:c.C661T:p.L221F	rs139913632

## Discussion

We report an exceptional and sustained response to chemotherapy in a young adult with myoepithelial carcinoma arising from the popliteal fossa with lymph node and pulmonary metastasis. While it is difficult to identify literature with a response rate for this rare malignancy or with a denominator of non-responders, there are at least case reports where chemotherapy regimens with broad activity have been attempted. Two such reports have shown either complete or partial response to carboplatin/paclitaxel [5, 6]. Partial response in two adult patients is briefly mentioned in a myoepithelial tumor review from 2008, whereas a second report details a patient with a metastatic vulvar mass. Diagnosis in this case was made based upon immunohistochemical staining profile, which similarly was notable for CAM5.2 and focal CKAE1/3 reactivity. The patient was treated initially with excision of the mass and bilateral inguinal lymph node dissection followed by pelvic radiation, but a broad chemotherapy regimen utilizing carboplatin and paclitaxel was initiated after the development of pulmonary metastasis. A complete pathologic response was noted and the patient remained in complete remission over 3 years. An additional study showed complete response to both ifosfamide and melphalan in a patient with metastatic soft tissue disease [19]. In this report, the patient initially presented with a tumor in their toe, which subsequently recurred in the ipsilateral lower extremity following radical disarticulation. Hyperthermic isolated limb perfusion using tumor necrosis factor and melphalan was initiated with a complete response. Pelvic metastases were later noted, successfully treated with ifosfamide and radiation. Chemotherapeutic response in the pediatric population is similarly difficult to identify. In a series of 29 pediatric patients with soft tissue myoepithelial carcinoma, approximately half received chemotherapy [1]. Of these, only one patient demonstrated a clinical response, seen after multiple cycles of doxorubicin and ifosfamide due to metastasis following initial tumor excision. An additional series of 7 non-metastatic, pediatric patients reported favorable outcomes following a regimen utilizing cisplatin with six patients remaining without evidence of disease at a mean follow-up of 2.5 years [20]. Three of the seven tumors had *EWSR1* rearrangement which was previously identified in series where it was associated with superficial location and more likely to be benign [12, 21]. Our sequencing methodology would be unlikely to have detected an *EWRS1* structural variation but clinical testing as mentioned above was negative.

Local surgical tumor excision with wide margins is recommended for myoepithelial carcinoma of soft tissues, although the optimal approach to treatment has yet to be established [6]. The efficacy of radiation or chemotherapy, either as an adjuvant therapy or in metastatic disease, also has not been consistently demonstrated [1, 6, 11]. As is common with rare malignancies, there is a lack of consensus guidelines and multiple options for care. In this case chemotherapy was incorporated because of the patients young age and metastatic presentation, but there are likely many scenarios whereby chemotherapy may be of benefit for patients with a similar malignancy whereby chemotherapy may not be considered. In fact, myoepithelial carcinoma is currently incorporated into a cooperative group clinical trial for soft tissue sarcomas as a chemotherapy resistant tumor eligible only for the “non-chemotherapy cohort” (NCT02180867).

Histologically, myoepithelial tumors often display a variety of cellular morphologies, making identification and diagnosis more difficult. The tumors may be composed exclusively of a single cell type, but are more frequently present as a combination of epithelioid, spindle cell, plasmacytoid or clear cell types [9]. Immunohistochemical staining serves as a key step in differentiating from similar appearing tumors. Myoepithelial carcinomas are generally positive for S-100, cytokeratin, epithelial membrane antigen (EMA) and α-smooth muscle actin [9]. The differential diagnosis include carcinoma, melanoma, epithelioid sarcoma, extraskeletal myxoid chondrosarcoma and chordoma. *EWSR1* gene rearrangement is identified in only 50% of the soft tissue myoepithlelial carcinoma [22].

Using whole exome sequencing, we examined our patient’s tumor for possible oncogenic variants that may help elucidate a mechanism for chemotherapy sensitivity. Given that matched germline DNA was not available for comparison, we expect many of variants uncovered to be rare or private germline mutations or passenger somatic mutations and therefore of little oncogenic consequence. We were able to identify, however, truncating mutations in *RB1* and *MED12* that are very likely to be somatic oncogenic drivers in this patient given the well-established role of these two genes as tumor suppressors across multiple tumor types. To our knowledge, this is the first description of inactivating mutation in either of these two genes reported in this cancer type.

The retinoblastoma protein (*RB1*) is one of the most frequently affected tumor suppressors across multiple cancer histologies and plays a critical role in regulation of cell cycle and apoptosis [23]. *RB1* pathway deregulation has been reported in various benign and malignant salivary tumors, including malignant myoepithelioma [24]. Interestingly, preclinical and clinical evidence in multiple cancer types suggest that RB1 expressional loss is associated with increased responsiveness to conventional chemotherapies [23]. Additionally, a recent genomic study in small cell lung cancer showed that presence of *RB1* inactivating mutation was highly predictive of good response to platinum-based chemotherapy [25]. Childhood retinoblastoma, almost invariably caused by either germline or somatic mutational inactivation of *RB1*, is also highly responsive to platinum-based chemotherapy [26]. Given this mounting evidence, we hypothesize that *RB1* mutation in our patient’s tumor predisposed to chemosensitivity. In contrast, loss of the RNA polymerase II mediator complex member *MED12* has been shown to induce drug resistance, particularly to tyrosine kinase inhibitor therapy, via activation of transforming growth factor B receptor signaling [27].

In summary, we report a case of myoepithelial carcinoma with a *RB1* inactivating mutation that experienced a dramatic response to platinum-based chemotherapy. We believe that our case adds to growing evidence across multiple cancer types that RB1 loss is predictive of chemosensitivity, perhaps in particular to platinum-based regimens. Given the rarity of this tumor type, the optimal systemic therapy approach is not well defined. Further study should be undertaken to evaluate whether RB1 loss is a recurring feature in this histology and whether platinum-based chemotherapy is more broadly effective in this tumor type outside of this case.

## Conclusion

While formal recommendations are difficult to make based on a case report, our review of the literature would suggest that continued consideration for systemic carcinoma therapy, more specifically with paclitaxel and carboplatin, should be considered in myoepithelial carcinoma patients presenting with stage 4 disease and extremity primary locations.

## Abbreviations

COSMIC: Catalogue of Somatic Mutations in Cancer
CT: Computed Tomography
ExAC: Exome Aggregation Consoritum
IRB: Institutional Review Board
MAF: Minor allele frequency
MRI: Magnetic Resonance Imaging
NCT: National Clinical Trial
NHLBI: National Heart, Lung, and Blood Institute
